# Topology-Induced Reduction in the Order–Disorder Transition in AB Block Copolymer: A Unit-Matched Comparison of Diblock, Multiblock, Comb, and Star Architectures

**DOI:** 10.3390/polym18070869

**Published:** 2026-04-01

**Authors:** June Huh

**Affiliations:** Department of Chemical and Biological Engineering, Korea University, Seoul 02841, Republic of Korea; junehuh@korea.ac.kr

**Keywords:** block copolymer, chain topology, order–disorder transition, directed self-assembly, graphoepitaxy, lamellae, alignment kinetics, defect annihilation, dynamical density functional theory

## Abstract

Chain topology offers a chemistry-preserving route to tune block copolymer (BCP) self-assembly by modifying intrachain correlations and relaxation pathways without changing monomer interactions. Here, we perform a unit-matched comparison of four lamella-forming AB architectures reconstructed from an identical constitutive diblock unit ($N_{0}$): a linear diblock (DB), a linear multiblock (MB), a comb-like architecture (CB), and a star-like architecture (SB). Using dynamical density functional theory (DDFT), we quantify topology-dependent bulk ordering thresholds and show that architectural reconfiguration systematically stabilizes the ordered phase, reducing the order–disorder transition relative to DB (MB/CB/SB ≈0.793/0.762/0.752 of the diblock value), in semi-quantitative agreement with random phase approximation (RPA) spinodal trends. We also compare topology-dependent directed self-assembly in a common trench geometry under matched reduced quench depth $\Delta \left(\chi N_{0}\right)=\chi N_{0}-{\left(\chi N_{0}\right)}_{\mathrm{ODT}}$, thereby isolating kinetic differences at comparable thermodynamic distance from bulk ordering. A Fourier-based alignment order parameter α(t) reveals sigmoidal alignment kinetics over decades in time and is well captured by a logistic form in lnt, enabling compact descriptors ($t_{50}$, $t_{90}$, and a steepness parameter *k*) that separate alignment onset from late-stage defect annihilation, while selective sidewalls robustly template sidewall-parallel lamellae across all topologies, the late-stage kinetics remain strongly connectivity dependent and can exhibit long-tailed completion associated with slow late-stage defect annihilation. These results demonstrate a dual role of topology in DSA: lowering the segregation strength required for bulk ordering while reshaping defect-mediated alignment pathways under confinement.

## 1. Introduction

Block copolymer (BCP) self-assembly offers a scalable route to periodic nanostructures with characteristic length scales beyond the practical reach of conventional top–down lithography. Because equilibrium microdomains can be formed with long-range periodicity, BCPs have become a central materials platform for nanopatterning and directed self-assembly (DSA) in advanced semiconductor manufacturing [1,2,3,4]. In such applications, the minimum attainable feature size is ultimately limited by the natural domain spacing (pitch) of the equilibrium morphology, which is set by chain dimensions and the thermodynamic driving force for segregation.

For a given monomer pair with a fixed Flory–Huggins interaction parameter χ, reducing the pitch generally requires decreasing the degree of polymerization *N*, since the characteristic spacing primarily scales with chain size. At the same time, microphase separation is only thermodynamically stable when the segregation strength χN exceeds the critical value at the order–disorder transition (ODT), ${\left(\chi N\right)}_{\mathrm{ODT}}$. This creates a fundamental trade-off for pitch scaling: while smaller *N* is preferred to shrink the equilibrium period, sufficiently large χN must be maintained to preserve ordered morphologies [5,6].

Historically, sub-10 nm BCP patterning has therefore relied predominantly on a “high-χ strategy,” in which new block chemistries with large χ are developed so that $\chi N>{\left(\chi N\right)}_{\mathrm{ODT}}$ even at small *N* [7,8,9,10,11,12,13,14,15,16,17,18,19,20,21]. Although effective, this strategy is often constrained by synthetic complexity, limited material choices, and integration challenges associated with introducing new chemistries into device processes. A complementary and comparatively less explored route is to engineer chain architecture such that ${\left(\chi N\right)}_{\mathrm{ODT}}$ itself is reduced, enabling ordering at a given χ with a smaller *N*—and thus a smaller pitch—without changing the chemical identity of the blocks [21,22,23,24]. In this view, the minimum chain length required for ordering can be estimated as $N_{\min}≃{\left(\chi N\right)}_{\mathrm{ODT}}/\chi$, implying that architectural designs that lower ${\left(\chi N\right)}_{\mathrm{ODT}}$ directly expand the accessible pitch window for non-high-χ material platforms.

For DSA, the practical question is not simply whether architecture affects ordering, but which connectivity enables microphase separation at the lowest segregation strength without changing chemistry. Although architecture-dependent shifts of the ODT have been reported [21,23,24], a topology-only benchmark remains scarce: in most studies, changing architecture simultaneously alters block chemistry, overall molecular size, or architecture-specific design parameters, obscuring the intrinsic effect of connectivity on ${\left(\chi N\right)}_{\mathrm{ODT}}$. Consequently, even for canonical AB architectures—diblock (DB), linear multiblock (MB), comb-like (CB), and star-like (SB) polymers—it is still unclear how their ${\left(\chi N\right)}_{\mathrm{ODT}}$ values rank when they are rebuilt from identical AB building blocks and examined under strictly unit-matched conditions.

Establishing such a unit-matched comparison is practically relevant because these topologies can, in principle, be generated by reconfiguring identical AB diblock building blocks while preserving chemical composition. A controlled ranking of ${\left(\chi N\right)}_{\mathrm{ODT}}$ would therefore quantify how much ordering promotion can be achieved by connectivity changes at fixed chemistry. Moreover, while topology-induced reductions in ${\left(\chi N\right)}_{\mathrm{ODT}}$ are most often discussed in terms of bulk thermodynamics, their implications for DSA—where confinement, defectivity, and ordering/alignment kinetics determine the process window—have been less systematically quantified.

In this work, we address these gaps by systematically investigating how chain topology, constructed from an identical constitutive AB diblock unit, influences both bulk ordering thermodynamics and graphoepitaxial assembly dynamics. Four representative architectures are considered: a linear diblock (DB) as a reference, a linear multiblock (MB), a comb-like architecture (CB), and a star-like architecture (SB). The constitutive diblock length $N_{0}$ and composition (f=0.5) are fixed, and the non-diblock architectures are formed by reconnecting the same AB building blocks, thereby isolating topological effects from chemical and compositional variations.

We first quantify topology-dependent shifts in the bulk ODT by determining ${\left(\chi N_{0}\right)}_{\mathrm{ODT}}$ for each architecture. To assess implications for DSA without conflating thermodynamic driving forces, we then perform graphoepitaxial trench simulations under $\Delta (\chi N_{0})$-matched conditions, where the quench depth $\Delta \left(\chi N_{0}\right)=\left(\chi N_{0}\right)-{\left(\chi N_{0}\right)}_{\mathrm{ODT}}$ is held constant across all topologies. This strategy ensures an equivalent thermodynamic distance from the respective ODTs, enabling a controlled comparison of ordering and alignment kinetics. Overall, the results demonstrate that chain topology provides a chemistry-preserving lever to reduce the ordering threshold and modulate graphoepitaxial kinetics, offering a practical pathway toward smaller-pitch, low-χ DSA through architectural reconfiguration rather than new material discovery.

## 2. Simulation Methods

We employed three-dimensional mesoscale simulations using the dynamic density functional theory (DDFT) [25,26] to compare the bulk order–disorder transition (ODT) and graphoepitaxial ordering kinetics of different chain topologies under $\Delta (\chi N_{0})$-matched conditions.

The polymer melt was modeled as a quasi-incompressible binary mixture of coarse-grained *A* and *B* beads, described by local density fields $\rho_{A}\left(r,t\right)$ and $\rho_{B}\left(r,t\right)$. Quasi-incompressibility was enforced via a compressibility penalty, such that the total density remains nearly constant,

(1) $$\rho_{A}\left(r,t\right)+\rho_{B}\left(r,t\right)\approx \rho_{0},$$

where $\rho_{0}$ is the reference total bead density. Under quasi-incompressible conditions, the compositional dynamics can be written in an exchange form driven by gradients of the chemical-potential difference. Specifically, we integrated

(2) $$\frac{\partial \rho_{A}}{\partial t}=M\nabla \cdot \left[\rho_{A}\rho_{B}\nabla \left(\mu_{A}-\mu_{B}\right)\right]+\eta \left(r,t\right),$$

where *M* is an effective mobility coefficient related to the bead diffusion coefficient via the Einstein relation ($D=Mk_{B}T$), and $\mu_{I}\left(r,t\right)=\delta F/\delta \rho_{I}\left(r,t\right)$ (I=A,B) are intrinsic chemical potentials derived from the free-energy functional F[ρ]. The stochastic term η(r,t) was taken as Gaussian noise consistent with the fluctuation–dissipation theorem.

The free energy functional was expressed as

(3) $$F\left[\rho \right]=F_{\mathrm{id}}\left[\rho \right]+F_{\mathrm{int}}\left[\rho \right]+F_{\mathrm{ext}}\left[\rho \right].$$

The ideal-chain contribution $F_{\mathrm{id}}$ captures intrachain correlations of Gaussian chains in self-consistent fields. Nonbonded interactions were modeled by a Flory–Huggins-type mean-field term

(4) $$F_{\mathrm{int}}\left[\rho \right]=\frac{1}{2}\sum_{I,J\in \{A,B\}}\int \;\;\int \epsilon_{IJ}\;\left(|r-r^{\prime }|\right)\;\rho_{I}\left(r\right)\rho_{J}\left(r^{\prime }\right)\;dr\;dr^{\prime },$$

where $\epsilon_{IJ}$ denotes the short-ranged interaction kernel between bead types *I* and *J*. The segregation strength is controlled through the effective Flory–Huggins interaction parameter χ, which is defined in terms of the interaction kernel as

(5) $$\chi =\frac{1}{k_{B}T}\left(\epsilon_{AB}-\frac{1}{2}\left[\epsilon_{AA}+\epsilon_{BB}\right]\right),$$

so that a positive χ corresponds to a net repulsive interaction between *A* and *B* beads. In the present study, $\epsilon_{AA}$ and $\epsilon_{BB}$ were fixed to zero, and χ was tuned by varying $\epsilon_{AB}$.

Confinement and surface interactions were incorporated through component-specific external potentials,

(6) $$F_{\mathrm{ext}}\left[\rho \right]=\sum_{I\in \{A,B\}}\int U_{I}\left(r\right)\;\rho_{I}\left(r\right)\;dr,$$

where $U_{I}\left(r\right)$ represents external fields associated with confining surfaces, including trench sidewalls, the substrate, and the film-top interface, acting on component *I*. Impenetrability was implemented using the built-in mask functionality, where a mask indicator m(r)∈{0,1} is defined such that m(r)=1 inside the polymer-accessible trench region and m(r)=0 in the solid wall region; consequently, polymer occupancy is restricted to the trench interior and the equilibrium bead densities vanish in the masked region.

To distinguish surface chemistry from purely geometric confinement, we considered selective sidewalls and neutral substrate/film-top interfaces. Using indicator functions $m_{w}\left(r\right)$ and $m_{n}\left(r\right)$ that localize the sidewall and neutral-interface regions, respectively, we parameterized the external field as

(7) $$U_{I}\left(r\right)=U^{\mathrm{hard}}\left(r\right)+\epsilon_{wI}\;m_{w}\left(r\right)+\epsilon_{nI}\;m_{n}\left(r\right),$$

where $U^{\mathrm{hard}}$ enforces impenetrability. Neutral interfaces were imposed by setting $\epsilon_{nA}=\epsilon_{nB}$, whereas sidewall selectivity was imposed by $\epsilon_{wA}\neq \epsilon_{wB}$.

**Figure 1:**
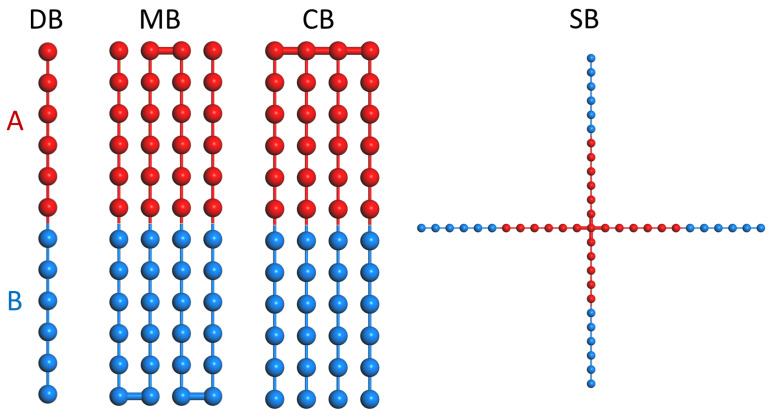
Schematic representations of the chain topologies considered in this study: diblock (DB), multiblock (MB), comb-like block (CB), and star-like block (SB). All architectures are constructed from identical symmetric AB diblock units ($N_{0}=12$, f=0.5). For MB, CB, and SB, four constitutive diblock units are connected into a single molecule in distinct topological arrangements, while DB serves as the linear reference. The thickened horizontal bonds in MB and CB indicate the covalent junction/connection points between constitutive AB units. Red and blue beads denote *A* and *B* segments, respectively.

All polymer architectures examined in this study are constructed from an identical constitutive AB diblock unit of length $N_{0}=12$ with symmetric composition (f=0.5), as illustrated in Figure 1. Four representative chain topologies are considered: diblock (DB), multiblock (MB), comb-like (CB), and star-like (SB). Except for the diblock control, all architectures contain the same number of constitutive diblock units (n=4), such that topological effects can be isolated from chemical composition and monomer-level interactions.

Bulk ODTs were determined from the steady-state squared composition order parameter $\langle \Psi^{2}\rangle$ as a function of $\chi N_{0}$, where the local composition deviation is defined as

(8) $$\Psi \left(r\right)=\frac{\rho_{A}\left(r\right)-\rho_{B}\left(r\right)}{\rho_{0}}.$$

Here, 〈·〉 denotes a spatial average over the simulation cell (with time-averaging over a steady-state window). The ODT location ${\left(\chi N_{0}\right)}_{\mathrm{ODT}}$ for each topology was extracted by fitting $\langle \Psi^{2}\rangle$–$\left(\chi N_{0}\right)$ to a sigmoidal (hyperbolic-tangent) form (see Section 3). The steady-state $\langle \Psi^{2}\rangle$ values were obtained by temporal averaging over the late-stage simulation window after the system reached a statistically steady regime. The apparent transition threshold was determined by fitting the $\langle \Psi^{2}\rangle$–$\chi N_{0}$ data to a sigmoidal function, and identifying the inflection point of the fit. The fitting was performed over the full range of $\chi N_{0}$ values.

Graphoepitaxial assembly was simulated by introducing three-dimensional trench geometries with selective sidewalls. The simulation domain was defined as a rectangular box with dimensions W×L×H=82×72×12a, where *a* denotes the grid spacing. Periodic boundary conditions were applied along the trench direction (*L*), while confinement was imposed in the transverse (*W*) and vertical (*H*) directions via impenetrable surfaces implemented through a mask function.

The bulk lamellar period, denoted by λ, is slightly topology-dependent (λ≈6.7a for DB and λ≈7.3a for MB/CB/SB), so that the trench dimensions correspond to W/λ≈11–12 and H/λ≈1.6–1.8 depending on the topology. This geometry therefore places the system in a common wide-trench regime laterally, while maintaining a thin-film thickness comparable to the natural domain spacing. The trench width was not separately optimized to achieve exact pitch commensurability for each topology; rather, a common geometry was used to compare architecture-dependent assembly under the same confinement condition. This geometry places the system in a wide-trench regime laterally while maintaining a thin-film thickness comparable to the natural domain spacing, thereby enabling a focused assessment of sidewall-guided ordering and alignment kinetics.

Surface chemistry in the trench simulations was implemented through the external potential $U_{I}\left(r\right)$ defined in Equation (7). Two sidewall-preference conditions were considered: an *A*-selective sidewall with $\left(\epsilon_{wA},\epsilon_{wB}\right)=\left(0,\epsilon_{AB}\right)$ and a *B*-selective sidewall with $\left(\epsilon_{wA},\epsilon_{wB}\right)=\left(\epsilon_{AB},0\right)$. For the symmetric diblock, these two cases are strictly equivalent under A/B exchange symmetry. For topologies that are not invariant under A/B exchange, additional *B*-selective tests confirmed that the qualitative alignment trends are insensitive to the choice of wall selectivity. Unless otherwise noted, results shown in the main text correspond to *A*-selective sidewalls.

To decouple thermodynamic driving forces from ordering kinetics, all trench simulations were performed under $\Delta (\chi N_{0})$-matched condition,

(9) $$\Delta \left(\chi N_{0}\right)=\left(\chi N_{0}\right)-{\left(\chi N_{0}\right)}_{\mathrm{ODT}},$$

such that each topology experiences the same reduced quench depth relative to its own bulk ODT.

The DDFT equations were solved on a three-dimensional Cartesian grid using an implicit Crank–Nicolson finite-difference scheme with a mixed density–potential solver. The dimensionless time step was set to 0.5, corresponding to a physical time step of 50ns for a bead diffusion coefficient of $D=1.0\times 10^{-7}\;{\mathrm{cm}}^{2}/s$. Each simulation was run for $2.0\times 10^{4}$ time steps, yielding a total physical time of 1.0ms. Numerical convergence was controlled using a solver tolerance of $10^{-3}$ with up to 100 iterations per step. The compressibility penalty parameter was fixed at $\kappa =20\;k_{B}T$, and the dimensionless noise parameter was set to 50. Time-resolved density fields were recorded every 50 steps for analysis.

## 3. Results

**Figure 2:**
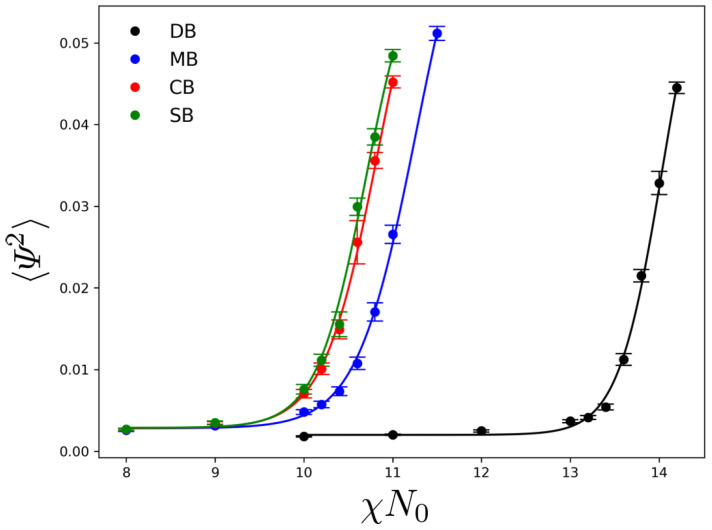
Steady-state squared order parameter $\langle \Psi^{2}\rangle$ as a function of $\chi N_{0}$ for DB, MB, CB, and SB. Symbols denote simulation data with error bars representing temporal fluctuations of the steady-state order parameter; solid lines are sigmoidal fits used to extract ${\left(\chi N_{0}\right)}_{\mathrm{ODT}}$.

Figure 2 shows the steady-state squared composition order parameter, $\langle \Psi^{2}\rangle$, as a function of the segregation strength $\chi N_{0}$ for the four architectures (DB, MB, CB, and SB). The local composition deviation is defined as $\Psi \left(r\right)=\left[\rho_{A}\left(r\right)-\rho_{B}\left(r\right)\right]/\rho_{0}$, and 〈·〉 denotes a spatial average over the simulation cell with additional time-averaging over a steady-state window. Error bars represent temporal fluctuations of $\langle \Psi^{2}\rangle$ in the steady state. In the low-$\chi N_{0}$ regime, all architectures exhibit a nearly constant baseline, $\langle \Psi^{2}\rangle ∼(2$–$3)\times 10^{-3}$, consistent with a disordered melt dominated by finite-amplitude composition fluctuations. Upon increasing $\chi N_{0}$, $\langle \Psi^{2}\rangle$ rises sharply, indicating the onset of sustained compositional modulation (i.e., microphase separation).

The apparent ordering threshold, ${\left(\chi N_{0}\right)}_{\mathrm{ODT}}$, was extracted from a sigmoidal fit of the steady-state $\langle \Psi^{2}\rangle$ as a function of $\chi N_{0}$ within the present stochastic DDFT protocol. To quantify the transition location, the $\langle \Psi^{2}\rangle$–$\left(\chi N_{0}\right)$ curves were fitted to a sigmoidal form,

(10) $$\langle \Psi^{2}\rangle =A+\frac{B}{2}\left[1+\tanh\left(\frac{\chi N_{0}-{\left(\chi N_{0}\right)}_{\mathrm{ODT}}}{w}\right)\right],$$

where ${\left(\chi N_{0}\right)}_{\mathrm{ODT}}$ corresponds to the inflection point and *w* captures the apparent transition width. A pronounced topology dependence is observed. DB requires substantially larger segregation strength to order, with the rapid increase occurring only at $\chi N_{0}≳13$ and a fitted value ${\left(\chi N_{0}\right)}_{\mathrm{ODT}}=14.1$. Notably, this value is significantly higher than the classical mean-field prediction for an infinite-*N* symmetric diblock, ${\left(\chi N\right)}_{\mathrm{ODT}}^{\mathrm{MF}}=10.495$ [5]. This upward shift is consistent with fluctuation and finite-chain-length effects, which renormalize the mean-field spinodal and shift the ODT upward [27]; in the present simulations, such effects are incorporated through the stochastic (noise-including) DDFT dynamics. In contrast, MB orders at intermediate segregation strength, ${\left(\chi N_{0}\right)}_{\mathrm{ODT}}=11.2$. CB and SB order at still lower segregation strengths, with ${\left(\chi N_{0}\right)}_{\mathrm{ODT}}=10.8$ (CB) and 10.6 (SB), indicating that these architectures stabilize ordered compositional modulation at substantially lower $\chi N_{0}$ than the diblock reference.

For reference, the mean-field spinodal was evaluated using the random phase approximation (RPA). Within RPA, the inverse structure factor is given by $1/S\left(x\right)=\sum_{\alpha \beta }G_{\alpha \beta }\left(x\right)/\left|G\left(x\right)\right|-2\chi$, where |G(x)| denotes the determinant of the 2×2 correlation matrix $G\left(x\right)=\{G_{\alpha \beta }\left(x\right)\}$, $x=q^{2}R^{2}$, *R* is the root-mean-square radius of gyration of a constitutive diblock, and $G_{\alpha \beta }\left(x\right)$ denotes the ideal single-chain density correlation functions. The spinodal condition follows from $1/S(x^{*})=0$ at the dominant wave vector $x^{*}$. The topology dependence enters solely through the architecture-specific forms of the single-chain correlation functions $G_{\alpha \beta }\left(x\right)$ (α,β=A/B), with $G_{AB}\left(x\right)=G_{BA}\left(x\right)$.

We introduce the following Gaussian-chain functions:

(11) $$g_{1}\left(x\right)=\frac{2}{x^{2}}\left[e^{-x/2}+\frac{x}{2}-1\right],\;g_{2}\left(x\right)=\frac{1}{x^{2}}{\left(1-e^{-x/2}\right)}^{2},$$

For DB:

(12) $$G_{AA}\left(x\right)=G_{BB}\left(x\right)=N_{0}g_{1}\left(x\right),\;G_{AB}\left(x\right)=N_{0}g_{2}\left(x\right).$$

For MB:

(13) $$\begin{array}{cc} G_{AA}\left(x\right) & =\frac{N_{0}}{4}\left[4g_{1}\left(x\right)+g_{2}\left(x\right)\left(2+4e^{-x}+4e^{-3x/2}+2e^{-3x}\right)\right],\\ G_{BB}\left(x\right) & =\frac{N_{0}}{4}\left[4g_{1}\left(x\right)+g_{2}\left(x\right)\left(4+2e^{-x}+4e^{-3x/2}+2e^{-2x}\right)\right],\\ G_{AB}\left(x\right) & =\frac{N_{0}}{4}g_{2}\left(x\right)\left(4+6e^{-x/2}+2e^{-x}+2e^{-2x}+2e^{-5x/2}\right). \end{array}$$

For CB:

(14) $$\begin{array}{cc} S(x) & =\sum_{i=1}^{4}\sum_{j\neq i}^{4}\exp\;\left[-\frac{x}{N_{0}}\left|i-j\right|\right],\\ G_{AA}\left(x\right) & =N_{0}\left[g_{1}\left(x\right)+\frac{S(x)}{4}g_{2}\left(x\right)\right],\\ G_{BB}\left(x\right) & =N_{0}\left[g_{1}\left(x\right)+\frac{S(x)}{4}g_{2}\left(x\right)e^{-x}\right],\\ G_{AB}\left(x\right) & =N_{0}g_{2}\left(x\right)\left(1+\frac{S(x)}{4}e^{-x/2}\right). \end{array}$$

For SB:

(15) $$\begin{array}{cc} G_{AA}\left(x\right) & =N_{0}\left[g_{1}\left(x\right)+3g_{2}\left(x\right)\right],\\ G_{BB}\left(x\right) & =N_{0}\left[g_{1}\left(x\right)+3g_{2}\left(x\right)e^{-x}\right],\\ G_{AB}\left(x\right) & =N_{0}g_{2}\left(x\right)\left(1+3e^{-x/2}\right). \end{array}$$

**Figure 3:**
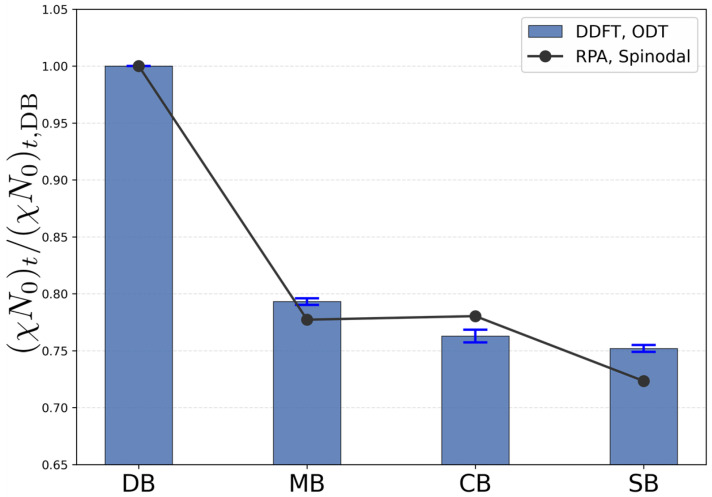
Normalized transition threshold ${\left(\chi N_{0}\right)}_{t}/{\left(\chi N_{0}\right)}_{t,\mathrm{DB}}$ for DB, MB, CB, and SB obtained from DDFT (bars) and RPA spinodal predictions (line with markers). All values are normalized by the diblock reference. Error bars from the DDFT fits are smaller than the symbol size and are omitted for clarity. The RPA results correspond to spinodal conditions and therefore represent a mean-field approximation to the ODT.

To isolate topology-induced shifts from the absolute fluctuation-induced renormalization of the ODT, the transition thresholds are normalized by the diblock reference, ${\left(\chi N_{0}\right)}_{t,\mathrm{DB}}$, and summarized in Figure 3. Within DDFT, MB, CB, and SB exhibit reductions to 0.793, 0.762, and 0.752 of the diblock value, respectively, confirming systematic stabilization of the ordered phase upon architectural reconfiguration.

For comparison, the corresponding RPA spinodal predictions yield normalized values of 0.78 (MB), 0.780 (CB), and 0.72 (SB). Despite the fact that RPA predicts the spinodal rather than the fluctuation-corrected ODT, the overall topology dependence is captured semi-quantitatively. In particular, both approaches consistently indicate that architectural reconfiguration reduces the segregation strength required for ordering relative to the diblock reference. Small quantitative discrepancies are expected due to the following reasons. For the symmetric diblock at f=0.5, the mean-field spinodal coincides with the ODT, whereas for MB, CB, and SB the architectures are not strictly invariant under *A*–*B* exchange, so the spinodal does not necessarily coincide with the true ODT even at the mean-field level. Furthermore, fluctuation effects beyond mean-field theory shift the DDFT ODT upward, while the RPA spinodal remains a mean-field prediction. Nevertheless, the close agreement in the relative reduction factors indicates that the primary architectural trend is robust and is mainly encoded in connectivity-driven modifications of intrachain correlations. In this sense, architecture-dependent segment-density distribution or chain-stretching effects are interpreted here as consequences of the altered connectivity rather than as an alternative mechanism independent of topology. At the level of Gaussian-chain mean-field thermodynamics, an equilibrium SCFT treatment would be expected to preserve the same qualitative topology ranking, because the leading architecture dependence is already encoded in the single-chain correlation functions entering the RPA kernel. Quantitative differences in the apparent transition values may nevertheless arise because the present thresholds are extracted from stochastic DDFT dynamics, whereas SCFT corresponds to an equilibrium saddle-point treatment.

To assess whether the ordering-promotion trend persists beyond the unit-matched n=4 benchmark, we additionally performed a higher-*n* sensitivity test for the comb-like architecture (CB, n=8). The apparent bulk ordering threshold is further reduced relative to DB and CB with n=4 (Figure S1).

Having established topology-dependent bulk ordering thresholds and their normalized trends (Figure 3), we next examine how the same set of architectures assemble under graphoepitaxial confinement when quenched to an identical reduced distance from the bulk transition, $\Delta \left(\chi N_{0}\right)=\chi N_{0}-{\left(\chi N_{0}\right)}_{\mathrm{ODT}}$. All simulations were performed in the same trench geometry (W≈11λ and H≈2λ), with *A*-selective sidewalls and otherwise neutral interfaces, so that differences in the ordering pathway reflect architecture-dependent kinetics under matched thermodynamic driving forces.

**Figure 4:**
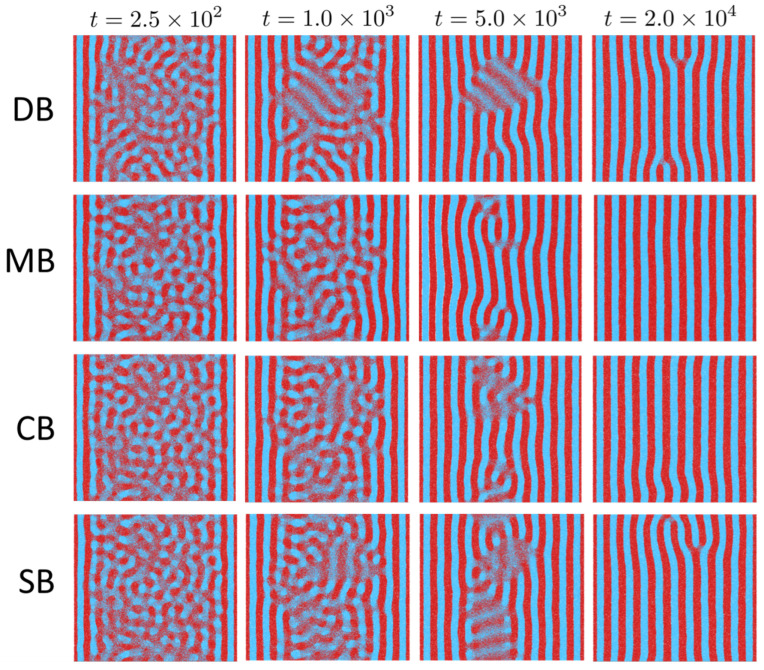
Time evolution of top-view graphoepitaxial lamellar morphologies in a trench for $\Delta \left(\chi N_{0}\right)=\chi N_{0}-{\left(\chi N_{0}\right)}_{\mathrm{ODT}}=3$. Rows correspond to chain topologies (DB, MB, CB, and SB) and columns correspond to simulation times $t=2.5\times 10^{2}$, $1.0\times 10^{3}$, $5.0\times 10^{3}$, and $2.0\times 10^{4}$. All simulations were performed in the same trench geometry (W≈11λ, H≈2λ) with selective sidewalls and otherwise neutral interfaces, so that differences reflect topology-dependent kinetics under a matched quench depth. Red and blue denote *A*- and *B*-rich domains, respectively.

Figure 4 shows representative top-view morphologies for $\Delta (\chi N_{0})=3$ at $t=2.5\times 10^{2}$, $10^{3}$, $5\times 10^{3}$, and $2\times 10^{4}$. At early times ($t=2.5\times 10^{2}$), all architectures remain largely disordered in the trench interior, while the selective sidewalls already bias the local composition and nucleate sidewall-parallel lamellar fragments. By $t=10^{3}$, these sidewall-templated lamellae extend further into the trench and the morphology transitions from short-range fluctuations to a partially aligned lamellar state. Subsequent evolution is dominated by coarsening and defect removal: by $t=5\times 10^{3}$ the patterns are largely sidewall-parallel but still contain one or a few extended dislocations/disclinations, typically localized near the trench centerline. This centerline localization is expected because the selective sidewalls template sidewall-parallel lamellae from both trench boundaries, while the trench center is the region least directly biased by the walls and the last place where the two inward-growing registries meet. Consequently, residual phase or spacing mismatch is preferentially accommodated near the trench centerline rather than at the strongly wall-pinned edges. By $t=2\times 10^{4}$, all four architectures approach a well-aligned lamellar state across the trench width, with residual distortions confined to localized defect cores. Qualitatively, the main topology dependence at this moderate quench depth is expressed in the persistence and shape of the remaining defects rather than in the global orientation imposed by the sidewalls.

Morphology evolutions in Figure 4 show that selective sidewalls reproducibly template sidewall-parallel lamellae across all architectures, while the late-stage evolution is dominated by defect rearrangement and annealing. The apparent kinetics and the persistence of trapped defect motifs vary with both chain connectivity. Here, the variation of ‘defect motifs’ does not mean that distinct topological defect classes emerge for different architectures. Rather, all architectures exhibit the same generic late-stage defects, primarily extended dislocations/disclinations near the trench centerline, but chain connectivity alters their spatial extent, shape, and persistence as they evolve toward localized defect cores. For MB, CB, and SB, which are not strictly invariant under A/B exchange, we also performed simulations with B-selective sidewalls. The resulting morphology evolution and kinetic trends were qualitatively similar to those of the A-selective case, with no additional defect motifs or distinct kinetic pathways observed.

Representative graphoepitaxial morphologies for CB with n=8 under matched $\Delta (\chi N_{0})$ remain sidewall-parallel, and the main visible difference is that they appear to retain somewhat more persistent residual defects rather than showing a qualitative change in the final aligned morphology (Figure S2).

While the morphology snapshots reveal clear topology-dependent differences in defect persistence at the present matched reduced quench depth, a more systematic comparison requires quantitative metrics. We therefore quantify the global alignment kinetics using a Fourier-based order parameter α(t) extracted from the time-resolved composition fields.

**Figure 5:**
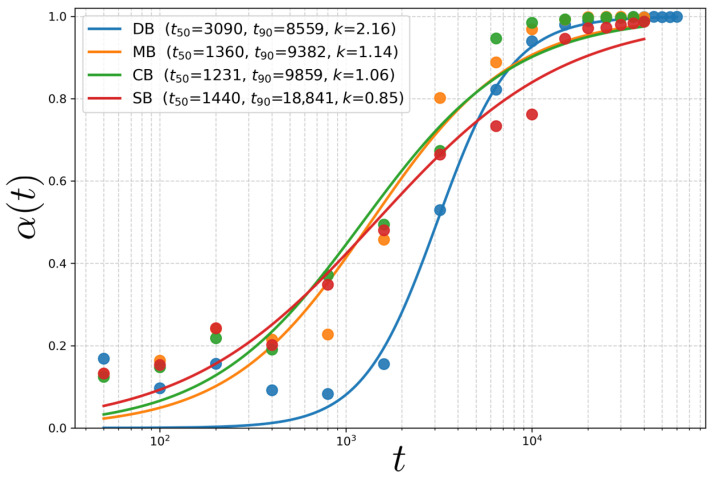
Time evolution of the alignment order parameter, α(t), for DB, MB, CB, and SB architectures at $\Delta (\chi N_{0})=3$. Symbols represent DDFT simulation data, while solid lines denote fits to a logistic function in logarithmic time. The characteristic times $t_{50}$ and $t_{90}$ correspond to the times at which α(t) reaches 0.5 and 0.9, respectively, and *k* denotes the steepness parameter of the log-time logistic fit. CB exhibits the earliest onset of alignment (smallest $t_{50}$), whereas DB shows the fastest completion (smallest $t_{90}$) due to its larger steepness *k*; SB remains the slowest to complete alignment (largest $t_{90}$).

Figure 5 shows the time evolution of the alignment order parameter α(t) for the four architectures and the corresponding fits to the log-time logistic form.

The lamellar alignment is quantified using a Fourier-based metric defined as

(16) $$\alpha \left(t\right)=\frac{I_{\|}\left(t\right)}{I_{\|}\left(t\right)+I_{\mathrm{np}}\left(t\right)}=\frac{I_{\|}\left(t\right)}{I_{\mathrm{tot}}\left(t\right)}.$$

Here Ψ(r,t) is the composition order-parameter field defined in Equation (8), and $\tilde{\Psi }\left(q,t\right)$ denotes its spatial Fourier transform. We define the spectral power (power spectrum) as

(17) $$P\left(q,t\right)={\left|\tilde{\Psi }\left(q,t\right)\right|}^{2}.$$

The wave vector q is expressed in polar coordinates (q,φ), where φ=0 is chosen to coincide with the target wave vector direction corresponding to sidewall-parallel lamellae (and φ=π accounts for the opposite wave vector due to the centrosymmetry of the Fourier spectrum). The spectral weights are evaluated by integrating P(q,φ,t) over the first-order annulus $q\in [q_{\min},q_{\max}]$ centered at $q^{*}≃2\pi /\lambda$:

(18) $$\begin{array}{cc} I_{\|}\left(t\right) & =\int_{q_{\min}}^{q_{\max}}dq\left[\int_{-\Delta \varphi }^{+\Delta \varphi }d\varphi \;P\left(q,\varphi ,t\right)+\int_{\pi -\Delta \varphi }^{\pi +\Delta \varphi }d\varphi \;P\left(q,\varphi ,t\right)\right], \end{array}$$

(19) $$\begin{array}{cc} I_{\mathrm{tot}}\left(t\right)\; & =\int_{q_{\min}}^{q_{\max}}dq\int_{0}^{2\pi }d\varphi \;P\left(q,\varphi ,t\right), \end{array}$$

(20) $$\begin{array}{cc} I_{\mathrm{np}}\left(t\right) & =I_{\mathrm{tot}}\left(t\right)-I_{\|}\left(t\right), \end{array}$$

with Δφ=π/12 unless otherwise noted. Because the integration is restricted to a narrow first-order annulus around $q^{*}$, the Jacobian factor *q* in $d^{2}q=q\;dq\;d\varphi$ does not appreciably affect the intensity ratio and is therefore omitted. In practice, the above integrals are evaluated as discrete sums on the FFT grid, and $q^{*}$ is identified as the dominant first-order peak of the radially averaged spectrum.

For all architectures, the alignment order parameter α(t) increases from a weakly biased state (α≪1) to a highly aligned state (α→1) and, when plotted against logarithmic time, exhibits a robust sigmoidal growth spanning multiple decades in *t*. Accordingly, we parameterize the alignment kinetics using a logistic function in lnt,

(21) $$\alpha \left(t\right)=\frac{1}{1+\exp\left[-k(\ln t-\ln t_{50})\right]},$$

where $t_{50}$ denotes the inflection time at which $\alpha (t_{50})=0.5$, and *k* controls the sharpness (steepness) of the transition on a logarithmic time axis. Equation (21) is equivalently written as a Hill-type form [28],

(22) $$\alpha \left(t\right)=\frac{{\left(t/t_{50}\right)}^{k}}{1+{\left(t/t_{50}\right)}^{k}},$$

which provides a compact descriptor for a bounded order parameter (0≤α≤1) evolving over broad timescales.

A simple rationale for the lnt-logistic form follows from an effective rate equation written in log-time,

(23) $$\frac{d\alpha }{d\ln t}=k\;\alpha \left(1-\alpha \right),$$

whose solution is Equation (21). This form is mathematically equivalent to the log-logistic (Hill-type) time-response function frequently used to describe sigmoidal kinetics [29]. In terms of physical time, the above equation corresponds to dα/dt=(k/t)α(1−α), i.e., an effective alignment rate that slows approximately as 1/t. Such progressive slowing is consistent with defect-mediated ordering in block copolymer thin films, where defect annihilation proceeds through collective rearrangements over finite free-energy barriers and becomes increasingly sluggish as defects become more dilute at late times [30,31,32]. The logarithmic time formulation effectively captures a broad distribution of activation timescales, as expected for defect annihilation processes governed by collective, barrier-crossing events. Within the present Gaussian-chain DDFT framework, this kinetic interpretation should be understood in terms of connectivity-dependent collective rearrangement barriers; explicit entanglement effects are not resolved.

From the fitted parameters, we further report $t_{90}$ as a practical completion time defined by $\alpha (t_{90})=0.9$, yielding

(24) $$t_{90}=t_{50}\exp\left(\frac{\ln9}{k}\right),$$

so that $t_{50}$ characterizes the onset/inflection of alignment while *k* (equivalently $t_{90}/t_{50}=9^{1/k}$) quantifies the width of the transition on a logarithmic time axis.

DB exhibits a delayed onset of global alignment (larger $t_{50}$) but a markedly sharper transition (largest *k*) and the fastest approach to near-complete alignment (smallest $t_{90}$). A plausible molecular interpretation is that DB, as the simplest linear topology without junction constraints and with a shorter overall chain length than the architectures reconstructed from four AB units (MB/CB/SB), can anneal extended defects more efficiently once a continuous sidewall-parallel registry spanning the trench is established. In this view, $t_{50}$ reflects the time required for a trench-spanning aligned pathway (or dominant grain) to emerge, whereas the subsequent steep rise in α(t) (large *k*) is governed by rapid defect-core rearrangement and annihilation enabled by minimal connectivity constraints.

MB and CB show intermediate behavior: both exhibit early onset of alignment (small $t_{50}$), but their smaller steepness parameters (k≈1.1) lead to broader transitions and $t_{90}$ values comparable to or slightly longer than DB ($t_{90}∼9–10\times 10^{3}$). This indicates a longer late-stage kinetic tail, consistent with slower defect annealing as the system approaches α→1.

By contrast, SB shows the broadest transition (smallest *k*) and the slowest completion (largest $t_{90}$), indicating sluggish late-stage defect removal. This behavior is consistent with star-like connectivity, in which a high-functionality central junction localizes connectivity constraints and thereby raises the barrier for the cooperative interfacial motions required for defect-core rearrangement, glide, and annihilation. However, we emphasize that this interpretation is not based on entanglement, as entanglement is not explicitly represented in the present Gaussian-chain DDFT framework.

## 4. Concluding Remarks

In this work, we established a topology-only benchmark for canonical AB block copolymer architectures by reconstructing diblock (DB), multiblock (MB), comb-like (CB), and star-like (SB) chains from an identical constitutive AB unit under strict, unit-matched conditions. By combining noise-including DDFT simulations with $\Delta (\chi N_{0})$-matched graphoepitaxial quenches, we decoupled thermodynamic driving force from kinetic response, thereby isolating the intrinsic role of chain connectivity in both bulk ordering and confined assembly dynamics.

Architectural reconfiguration alone substantially reduces the bulk ordering threshold relative to the diblock reference. MB, CB, and SB consistently exhibit lower ${\left(\chi N_{0}\right)}_{\mathrm{ODT}}$ values than DB, and the relative reductions are captured semi-quantitatively by RPA spinodal analysis. This confirms that the primary effect of topology is encoded in connectivity-driven modifications of intrachain correlation functions. From a materials-design perspective, lowering ${\left(\chi N_{0}\right)}_{\mathrm{ODT}}$ through architecture provides a chemistry-preserving route to enable ordered morphologies at smaller *N*, thereby expanding the accessible pitch window without invoking high-χ strategies.

Under graphoepitaxial confinement at a matched, reduced quench depth, all architectures reproducibly form sidewall-parallel lamellae, yet their ordering pathways and late-stage defect-annihilation kinetics remain strongly topology-dependent. The Fourier-based alignment descriptor α(t), parameterized by a log-time logistic form, provides compact kinetic metrics ($t_{50}$, $t_{90}$, and *k*) that separate the onset of global alignment from the completion of defect removal. At moderate quench depth, topology primarily influences the persistence and morphology of extended defects, whereas the global orientation itself is robustly templated by the sidewalls. Notably, the $\Delta (\chi N_{0})$-matched comparison shows that a lower bulk ordering threshold does not automatically imply faster graphoepitaxial perfection under confinement. At the present moderate reduced quench depth ($\Delta (\chi N_{0})=3$), all architectures reach strong sidewall-parallel alignment, yet the kinetic descriptors separate the onset of global alignment from the completion of defect removal: CB exhibits the earliest onset (smallest $t_{50}$), whereas DB completes most rapidly (smallest $t_{90}$) due to its steeper log-time transition, and SB shows the slowest completion, consistent with junction-localized constraints that hinder late-stage defect glide and annihilation. This onset–completion decoupling highlights the importance of reporting both $t_{50}$ and $t_{90}$ (or *k*) when evaluating DSA performance across architectures at comparable thermodynamic distance from bulk ordering.

The bulk ordering-promotion trend is also expected to be qualitatively robust with respect to simulation framework. In particular, prior particle-based DPD results for related linked-diblock architectures support the same tendency toward reduced ordering thresholds with increasing connectivity, while the domain spacing changes only weakly. By contrast, the quantitative kinetics of confined ordering are expected to be more framework dependent, since standard equilibrium SCFT does not resolve dynamics and particle-based simulations can alter absolute timescales and local defect-core structure.

The present unit-matched comparison demonstrates that chain topology provides two complementary design levers for directed self-assembly: a thermodynamic lever via reduction in the bulk ordering threshold and a kinetic lever via topology-dependent defect-annihilation pathways. The $\Delta (\chi N_{0})$-matched framework and log-time kinetic descriptors introduced here offer a general methodology for quantitatively assessing architectural strategies for pitch scaling while explicitly accounting for the potential risk of kinetic trapping in more demanding process conditions. Extending this topology-only benchmark to broader architectural families, alternative guiding geometries, and annealing protocols will further clarify how connectivity engineering can be translated into robust, defect-tolerant nanomanufacturing process windows.

## Data Availability

The data presented in this study are available on request from the corresponding author.

## Supplementary Materials

The following supporting information can be downloaded at: https://www.mdpi.com/article/10.3390/polym18070869/s1, Figure S1: Steady-state squared composition order parameter $\langle \Psi^{2}\rangle$ as a function of $\chi N_{0}$ for DB (n=1), CB with n=4, and CB with n=8; Figure S2: Representative time evolution of top-view graphoepitaxial lamellar morphologies for CB with n=4 and n=8 at $\Delta (\chi N_{0})=3$.
