# Supplementary material for: Topology-Induced Reduction in the Order–Disorder Transition in AB Block Copolymer: A Unit-Matched Comparison of Diblock, Multiblock, Comb, and Star Architectures
Source: Polymers (Basel). 2026 Apr 1;18(7):869. doi: 10.3390/polym18070869 (PMC13074441; doi:10.3390/polym18070869)
Supplement: Supplementary file 1 [file polymers-18-00869-s001.zip › polymers-4200020-supplementary.pdf]

Supporting Information

Topology-Induced Reduction of the Order–Disorder Transition in AB Block Copolymers: A Unit-Matched Comparison of Diblock, Multiblock, Comb, and Star Architectures

June Huh
Department of Chemical and Biological Engineering,
Korea University, Seoul 02841, Korea
junehuh@korea.ac.kr

Additional higher-n sensitivity test for the comb-like architecture

To assess whether the ordering-promotion trend persists beyond the unit-matched $n = 4$ benchmark used in the main text, we performed additional simulations for a comb-like (CB) architecture with an increased number of constitutive AB diblock units ($n = 8$). In the main manuscript, the non-diblock architectures were compared at fixed $n = 4$ so that the primary comparison isolates topology under identical constitutive-unit conditions. Here, CB was used as a feasible higher-n branched test case within the present simulation framework.

Figure S1 shows the steady-state squared composition order parameter $\langle \Psi^2 \rangle$ as a function of χN_0 for DB ($n = 1$), CB with $n = 4$, and CB with $n = 8$. The $\langle \Psi^2 \rangle$–χN_0 curve shifts systematically to lower χN_0 as n increases, indicating a monotonic reduction of the apparent ordering threshold with increasing branch number in the CB architecture. Thus, the bulk ordering-promotion trend identified in the main text remains operative beyond the $n = 4$ benchmark. This trend is also consistent with our previous study [1], which showed that increasing the number of linked diblock units lowers the ODT while altering the domain spacing only modestly.

To examine whether this higher-n effect changes the directed self-assembly pathway qualitatively, we also performed representative graphoepitaxial simulations for CB with $n = 8$ under the same trench geometry and the same matched reduced quench depth, $\Delta(\chi N_0) = 3$, as used in the main text. The resulting morphology evolution is compared with that of CB with $n = 4$ in Figure S2. In both cases, selective sidewalls first nucleate sidewall-parallel lamellar fragments near the trench boundaries, which then propagate into the trench interior and evolve toward a laterally aligned lamellar state. The main visible differences are expressed not in the global alignment direction itself, but in the persistence and shape of the remaining central defects during the intermediate and late stages. In particular, the higher-n CB case shows a slightly more persistent residual defect at late times, while still converging to the same qualitative sidewall-parallel lamellar morphology.

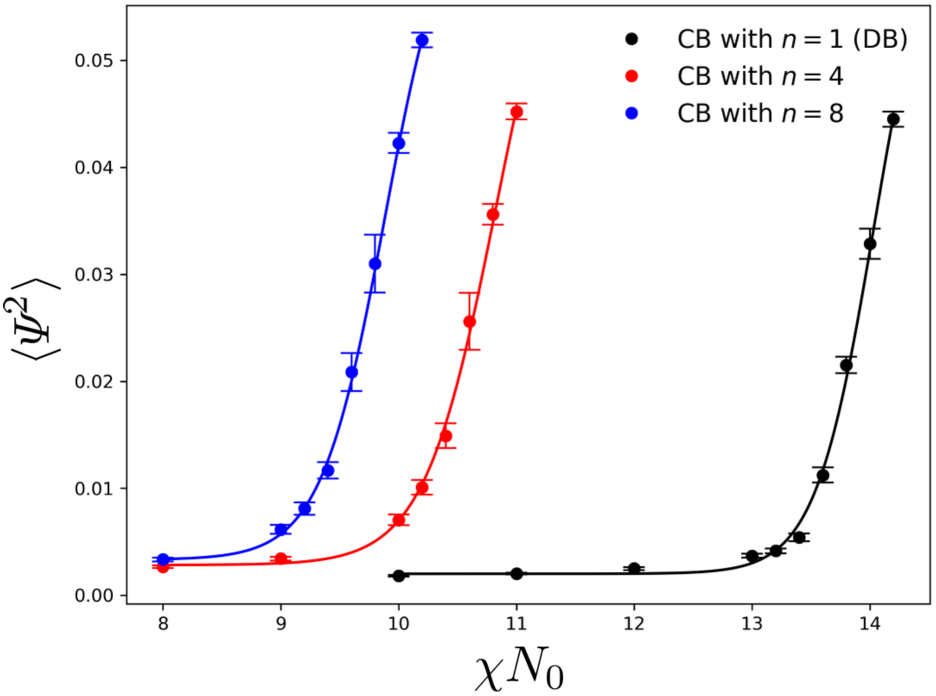

Figure 1: Steady-state squared composition order parameter $\langle \Psi^2 \rangle$ as a function of χN_0 for DB ($n = 1$), CB with $n = 4$, and CB with $n = 8$. Symbols denote simulation data with error bars representing steady-state fluctuations, and solid lines are sigmoidal fits used to extract the apparent ordering threshold. Increasing n in the comb-like architecture shifts the transition systematically to lower χN_0, indicating further promotion of bulk ordering.

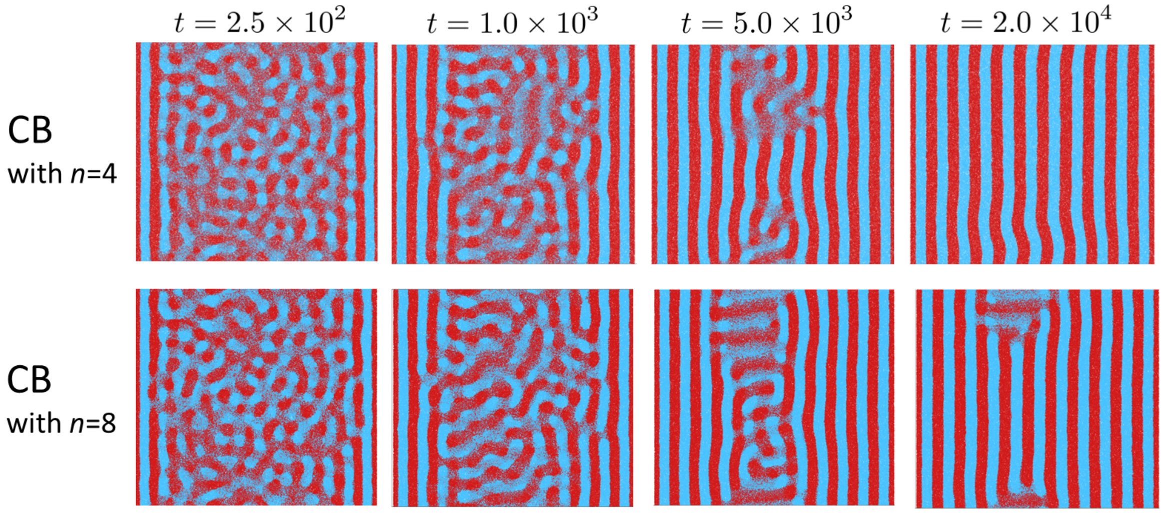

Figure 2: Representative time evolution of top-view graphoepitaxial lamellar morphologies for CB with $n = 4$ and $n = 8$ at $\Delta(\chi N_0) = 3$. Columns correspond to simulation times $t = 2.5 \times 10^2$, 1.0×10^3, 5.0×10^3, and 2.0×10^4. In both cases, selective sidewalls template sidewall-parallel lamellae, and the dominant visible difference is the persistence of residual defects near the trench centerline at intermediate and late times. Red and blue denote A- and B-rich domains, respectively.

References

[1] Park, J.; Shin, H.-W.; Bang, J.; Huh, J. Optimizing Chain Topology of Bottle Brush Copolymer for Promoting the Disorder-to-Order Transition. *Int. J. Mol. Sci.* **2022**, *23*, 5374.
